# Bortezomib alters sour taste sensitivity in mice

**DOI:** 10.1016/j.toxrep.2017.03.003

**Published:** 2017-03-10

**Authors:** Akihiro Ohishi, Kentaro Nishida, Karin Miyamoto, Mizuka Imai, Ryoko Nakanishi, Kyoko Kobayashi, Akiko Hayashi, Kazuki Nagasawa

**Affiliations:** Department of Environmental Biochemistry, Kyoto Pharmaceutical University 5 Nakauchi-cho, Misasagi, Yamashina-ku, Kyoto 607-8414, Japan

**Keywords:** AADC, aromatic amino acid decarboxylase, BSA, bovine serum albumin, CP, circumvallate papillae, DMSO, dimethyl sulfoxide, HCl, hydrochloric acid, HE, hematoxylin-eosin, MSG, mono sodium glutamate, NaCl, sodium chloride, QHCl, quinine hydrochloride, Taste disorder, Bortezomib, Sour taste, Chemotherapy, Adverse effect

## Abstract

Chemotherapy-induced taste disorder is one of the critical issues in cancer therapy. Bortezomib, a proteasome inhibitor, is a key agent in multiple myeloma therapy, but it induces a taste disorder. In this study, we investigated the characteristics of bortezomib-induced taste disorder and the underlying mechanism in mice. Among the five basic tastes, the sour taste sensitivity of mice was significantly increased by bortezomib administration. In bortezomib-administered mice, protein expression of PKD2L1 was increased. The increased sour taste sensitivity induced by bortezomib returned to the control level on cessation of its administration. These results suggest that an increase in protein expression of PKD2L1 enhances the sour taste sensitivity in bortezomib-administered mice, and this alteration is reversed on cessation of its administration.

## Highlights

• Bortezomib administration increase sour taste sensitivity in mice.
• Protein expression of PKD2L1 increases in circumvallate papillae of bortezomib-administered mice.
• Increased sour taste sensitivity induced by bortezomib returned to the control level on cessation of its administration.

## 1 Introduction

In cancer therapy, the administration of anticancer drugs has several adverse effects on patients. Recently, the mechanisms underlying some of them such as nausea and vomiting have been clarified, and thus effective methods for prevention or amelioration of them have been used in clinical situations [1], [2]. Taste disorder is one of the anticancer drug-related adverse effects, but some medical staffs are little concerned with it, because taste disorder is considered to have no or only a negligible effect on the outcome of chemotherapy. However, since taste sensing is critical for satisfying patients’ appetites through enjoyment of delicious food, dysfunction of taste reception induces malnutrition and decreases the quality of life of patients, leading to deterioration of their physiological condition and decreased motivation to fight against diseases [3]. In clinical situations, zinc supplementation is sometimes prescribed for patients, because zinc deficiency is known to cause taste disorder, but its effectiveness is not adequate [4]. Therefore, establishment of a protocol for prevention and amelioration of anticancer drug-induced taste disorder has been desired, but there is little available information of the underlying mechanism.

In general, anticancer drugs exhibit cytotoxicity and thus it is reasonable that they induce taste disorder through a decrease in the number of taste cells, on which taste receptors are expressed. In fact, Mukherjee et al. demonstrated that alkylating agent cyclophosphamide morphologically disrupts taste buds with a decrease in the number of taste cells in mice, resulting in decreases in their sweet and umami taste sensitivities [5], [6]. On the other hand, we recently demonstrated that a platinum anticancer drug, oxaliplatin, induces a decrease in sweet taste sensitivity in rats, which is due, at least in part, to alteration of the expression of a sweet taste receptor, T1R2, without any changes in the morphology of taste buds or the number of taste cells [7]. These findings imply that the characteristics of anticancer drug-induced taste disorder might differ with its pharmacological properties.

Bortezomib is a proteasome inhibitor [8] and is used for treatment of multiple myeloma [9]. As one of its adverse effects, taste disorder is reported to develop in patients [10], but its characteristics and the underlying mechanism remain unknown. In this study, therefore, to characterize bortezomib-induced taste disorder, we examined behavioral and histological alterations in the taste sensing and reception systems, respectively, in mice. Here, we found that bortezomib administration induced an increase in sour taste sensitivity and that this alteration was reversed on cessation of its administration. These changes correlate with protein expression of PKD2L1 but not the morphology of taste buds, number of taste cells, or expression levels of mRNA of PKD1L3 and PKD2L1.

## 2 Material and methods

### 2.1 Chemicals

Bortezomib (Catalog number: B-1408) was purchased from LC Laboratories (Boston, MA). Collagenase D (Catalog number: 1088858) and dispase II (Catalog number: 4942078) were purchased from Roche Applied Science (Tokyo, Japan). A trypsin inhibitor (Catalog number: T9128) and bovine serum albumin were purchased from Sigma-Aldrich (St. Louis, MO). All other chemicals and reagents were obtained from Wako Pure Chemical Ind. (Osaka, Japan), except where otherwise noted.

### 2.2 Animals and treatment

All experiments were approved by the Experimental Animal Research Committee of Kyoto Pharmaceutical University (authorization number: 13-12-005), and were performed according to the Guidelines for Animal Experimentation of Kyoto Pharmaceutical University. C57BL/6NCrl mice (25-wk-old; Charles River, Yokohama, Japan) were housed with food and water available *ad libitum* in a controlled environment with a 12 h/12 h light/dark cycle. Bortezomib was dissolved in dimethyl sulfoxide (DMSO) and was diluted to a concentration of 0.1 mg/mL with sterile saline (final concentration of DMSO in bortezomib solution was 0.5%). Bortezomib was administered subcutaneously (*s.c.*) to mice at a dose of 1 mg/kg body weight. Previous papers on bortezomib-induced neurotoxicity in mice reported that development of symptoms needed 1 or more week, and that the severity worsened with multiple administration [11], [12]. It is considered that this multiple administration-induced adverse effect of bortezomib might reflect its pharmacokinetic characteristics that the plasma half life ranged between 10 and 31 h, and was prolonged by its multiple administration [13], [14]. Thus, based on these information, we administered bortezomib on days 1, 4, 8, 11, 15, 18, 22, and 25 (total dose; 8 mg/kg). In the experiment in Fig. 5, bortezomib was administered on days 29, 32 and 36 additionally. In control mice, the same volume of sterile saline containing 0.5% DMSO was injected subcutaneously with the same dosing schedule.

### 2.3 Brief-access test

We adopted the brief-access test to evaluate the effects of bortezomib on behavioral responses to tastants in mice. Mice were randomly divided into 2 groups, control and bortezomib ones. All training and test sessions were performed during the light phase of the light/dark cycle. The mice had restricted access to water for more than 22 h before each training or test session. A training session was performed to obtain a stable lick number, and was performed for 6 days before initiation of bortezomib administrations and test sessions. On the first day of a training session, so that it would get used to the experimental apparatus, a mouse was placed in a test box (a black box to shield from light; 24 cm width, 17 cm depth and 11.5 cm height) and was given free-access to distilled water for 15 min from a polypropylene tube *via* an elliptical window (major axis: 15 mm, minor axis: 10 mm). A lick counter system (DELICIOUS; INECK, Kyoto, Japan) was set up between the edge of the tube and the window, and the lick number was determined automatically by recording the number of interceptions of the sensor beam by the tongue of a mouse when it licked the solution from the edge of the tube. On the second day of the training session, the mouse was trained to drink distilled water with a 10-s go/15-s no-go schedule, consisting of a 10 s-period of presentation of distilled water and a 15 s inter-presentation interval, this schedule being repeated 15–25 times. On the third and fourth days, the training was performed with the same procedure as that for a test session described below except for the use of distilled water instead of a taste solution. On the fifth or sixth day, the training was performed with the same procedure as that for the test session (Fig. S1), the lick numbers obtained in this session being used as the baseline data. To avoid excessive water deprivation, mice were allowed to drink water *ad libitum* for 1 h after the training on each day. After a 22 h-water deprivation period, each mouse was subjected to the test session, and the lick numbers were determined on 2, 5, 9, 12, 16, 19, 23, and 26 days after initiation of bortezomib administration. We performed two series of brief-access test which involved 1) five basic taste solutions and 2) sour taste solutions, as described below.

### 2.4 Five basic taste test

The sensitivity to the five basic tastes was assessed using 5 taste solutions. First, the lick number in 10 s for distilled water was determined, followed by a 15 s inter-presentation interval, and then the taste solution was presented for 10 s, followed by 5 s water presentation. Data are expressed as lick ratios as a quantitative index of taste sensitivity, and were calculated by dividing the lick number in 10 s for a taste solution by that for distilled water, because the lick number is affected by differences in motivation to drink solutions among mice. The number of licks of water used for lick ratio calculation was recorded preceding a trial with the first taste solution of each tastant. Taste solutions were presented in the order of sweet (300 and 50 mM sucrose with 0.3 mM quinine hydrochloride (QHCl)), sour (5 and 10 mM citric acid), bitter (0.3 and 1 mM QHCl), salty (150 and 500 mM sodium chloride (NaCl)), and umami (300 and 50 mM mono sodium glutamate (MSG) with 0.3 mM QHCl and 30 μM amiloride). The use of a mixture of sweet or umami and bitter taste solutions has been reported to allow sensitive detection of changes in these taste sensitivities [15], [16]. Because sweet and umami are palatable tastes while bitter is an unpalatable one for mice, on addition of the bitter tastant to the sweet or umami solution, the mice avoid drinking the mixture because it becomes hard for them to perceive the sweet or umami taste. Amiloride was added to the umami taste solution to reduce the amiloride-sensitive component of the salty taste [17]. The sweet and umami taste solutions were presented in descending order of their concentrations, while the sour, bitter, and salty taste ones were presented in ascending order. We excluded the data when mice could not accomplish a series of lick tests in each session of taste.

### 2.5 Sour taste test

This test was performed using 2 types of sour taste solutions (citric acid or hydrochloric acid (HCl)) to confirm the change in sour taste sensitivity in bortezomib-administered mice, and the effect of each sour tastant was examined with separate groups of mice. The taste solutions used in the experiments were prepared in the concentration ranges of 5–100 mM citric acid and 3.2–32 mM HCl (Supplementary Table 1). The series of solutions were presented in ascending order of sour tastant concentration. During each test session, the taste solution and distilled water were alternatively presented to a mouse for 10 and 5 s, respectively, distilled water being used to rinse the oral cavity. To compare the sour taste sensitivity between control and bortezomib-administered mice, we estimated the IC_50_ values for concentration-dependent alteration of the lick ratios for each mouse by non-linear curve fitting using GraphPad Prism Software version 6 (GraphPad Software, Inc., La Jolla, CA). We excluded the data when mice could not accomplish a series of lick tests on one day.

### 2.6 Exfoliation of epithelial tissue of circumvallate papillae (CP)

Mice were perfused transcardially with saline under deep anesthesia (pentobarbital sodium, 25 mg/kg, *i.p.*). As reported previously [18], mouse lingual epithelial tissue was exfoliated from the tongue by injection of an enzyme cocktail comprising 2.5 mg/mL dispase II, 1.0 mg/mL collagenase D, and 1.0 mg/mL trypsin inhibitor for 20 min at room temperture, and then the epithelial tissue was peeled off. After trimming of the outer region of CP, the tissue was treated with an RNAlater^®^ solution (Sigma-Aldrich) and kept at −80° C until use.

### 2.7 **Reverse transcription and real-time quantitative polymerase chain reaction (PCR) analyses**

Total RNA was extracted and reverse transcribed with a NucleoSpin RNA^®^ XS kit (Macherey-Nagel, Düren, Germany), and a PrimeScript™ RT reagent kit with gDNA Eraser (Takara, Shiga, Japan), respectively, according to the manufacturers’ instruction manuals. Real-time quantitative PCR was conducted with an ABI PRISM 7500 Real-time PCR System (Life Technologies, Tokyo, Japan) using SYBR Premix Ex Taq (Takara). The primer sets are shown in Table 1. All reactions for real-time quantitative PCR were carried out with the following parameters: 94 °C for 5 min, and 40 cycles of 94 °C for 30 s, 60 °C for 60 s, and 72 °C for 15 s. Negative control experiments were performed using DNase-free water instead of the template DNA, and specific amplification was confirmed using dissociation curves.

**Table 1:** Primers used for real-time quantitative PCR.

Gene		Primer sequences	Product size	Accession #
Pkd1l3 (PKD1L3)	F	5′-GCCTGTTCAGATGGTTGAAGTG-3′	116 bp	NM_181544
	R	5′-GCTGGTGGCTTGGTCTTTG-3′		

Pkd2l1 (PKD2L1)	F	5′-TCATTGTGGGCTGTGAAGTTG-3′	96 bp	NM_181422
	R	5′-TGAGGTAGCGAAGCCGATG-3′		

β-actin	F	5′- AGGTCATCACTATTGGCAACGA-3′	171 bp	NM_007393
	R	5′- CACTTCATGATGGAATTGAATGTAGTT-3′		

### 2.8 Tissue preparation for frozen sections

Mice were perfused transcardially with 4% paraformaldehyde in 0.1 M phosphate buffer (pH 7.4) containing 0.2% picric acid under deep anesthesia (pentobarbital sodium, 25 mg/kg, *i.p.*), and then their tongues, which contained CP, were picked up. The tongues were sectioned at 20 μm thickness with a freezing microtome (Leica CM1850; Leica, Nussloch, Germany), and then the sections were subjected to immunohistochemistry and hematoxylin-eosin (HE) staining.

### 2.9 Immunohistochemical analysis

The expression levels of antigens were determined by free-floating immunohistochemistry [18]. The free-floating sections were immunoreacted with the primary antibodies (Table 2) in PBS containing 1% donkey serum, 0.3% Triton-X-100, 0.3% bovine serum albumin and 0.05% sodium azide for 3 days at 4 °C, followed by incubation for 1 day at 4 °C with the secondary antibodies (Table 2) in the same buffer as that for the primary antibodies. For all immunostaining, a negative control, which was prepared by omitting the primary antibodies, was prepared, and the reproducibility of immunostaining was confirmed by assessing sections from 3 or 4 mice per immunostaining. The sections were mounted on glass slides and then enclosed using a Prolong^®^ antifade kit (Life Technologies). Photomicrographs were obtained under a confocal laser microscope (LSM510META; Carl Zeiss, Jena, Germany). Fluorescence intensity was measured using the histogram program of the Photoshop software (Adobe Systems, San Jose, CA).

**Table 2:** Antibodies used for immunohistochemistry.

Antigen	Primary Ab	Secondary Ab
PKD2L1	Rabbit polyclonal Ab (1:1000; OSP00055W, Osenses)	Donkey anti-rabbit IgG conjugated with Alexa Fluor^®^ 488 (1:1000; A21206, Life Technologies)
AADC	Rabbit polyclonal Ab (1:200; BML-AZ1030-0050, Enzo)	Donkey anti-rabbit IgG conjugated with Alexa Fluor^®^ 488 (1:1000; A21206, Life Technologies)
Car4	Goat polyclonal Ab (1:100; AF2414, R&D Systems)	Donkey anti-goat IgG conjugated with Alexa Fluor^®^ 594 (1:1000; A11016, Life Technologies)

### 2.10 Statistical analysis

**Fig. 1:**
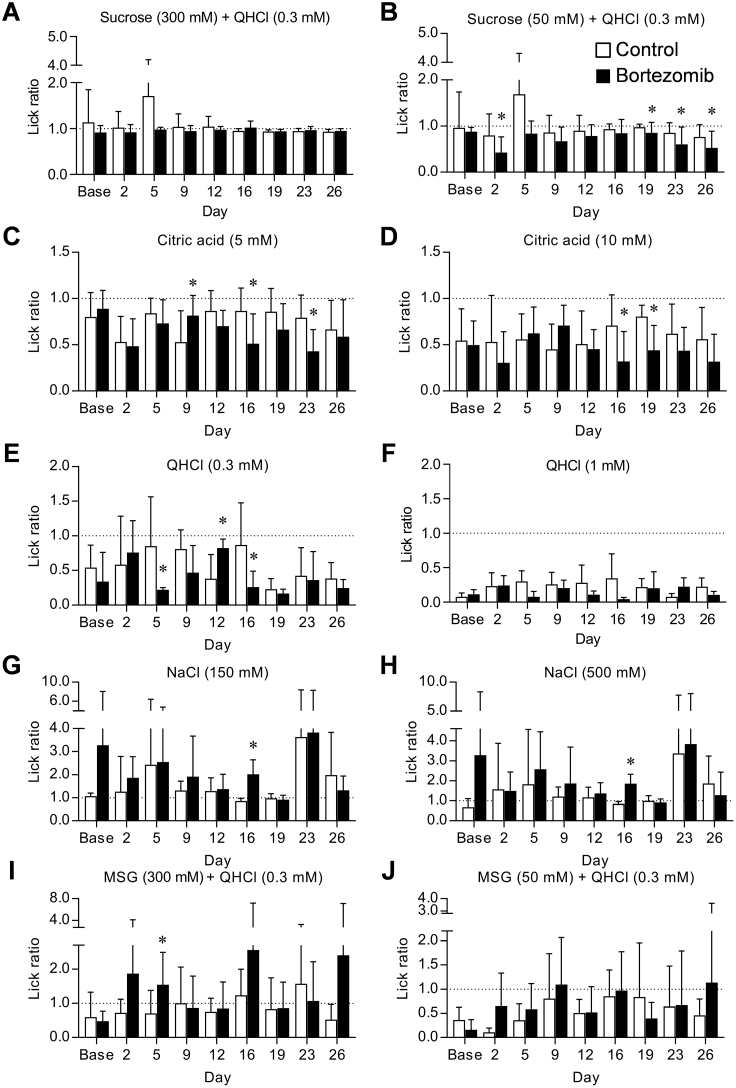
Behavioral responses to five basic taste solutions in bortezomib-administered mice. The lick ratios for 300 and 50 mM sucrose with 0.3 mM QHCl (A, B), 5 and 10 mM citric acid (C, D), 0.3 and 1 mM QHCl (E, F), 150 and 500 mM NaCl (G, H), and 300 and 50 mM MSG with 0.3 mM QHCl and 30 μM amiloride (I, J) were determined in control and bortezomib-administered mice on days 0 (Base), 2, 5, 9, 12, 16, 19, 23 and 26. Each bar represents the mean ± SD (N = 4–10). *: *p* <0.05 vs control in multiple comparison of Bonferroni test.

**Fig. 2:**
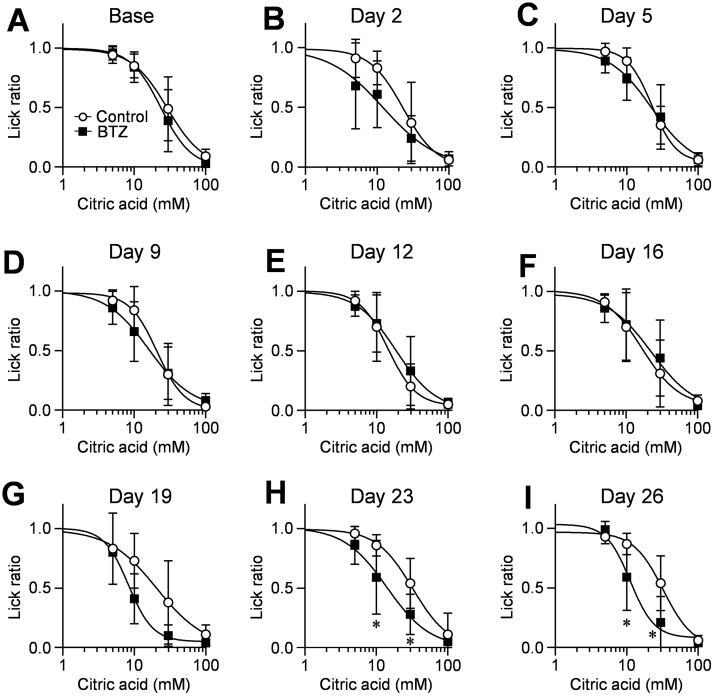
Behavioral responses to citric acid solutions in bortezomib-administered mice. The lick ratios for 5–100 mM citric acid (A–I) were determined in control and bortezomib-administered mice on days 0 (Base), 2, 5, 9, 12, 16, 19, 23 and 26. Concentrations of citric acid solutions were 5, 10, 30 and 100 mM. Each point represents the mean ± SD (N = 4–9). *: *p* <0.05 vs control in multiple comparison of Bonferroni test. BTZ: bortezomib.

**Fig. 3:**
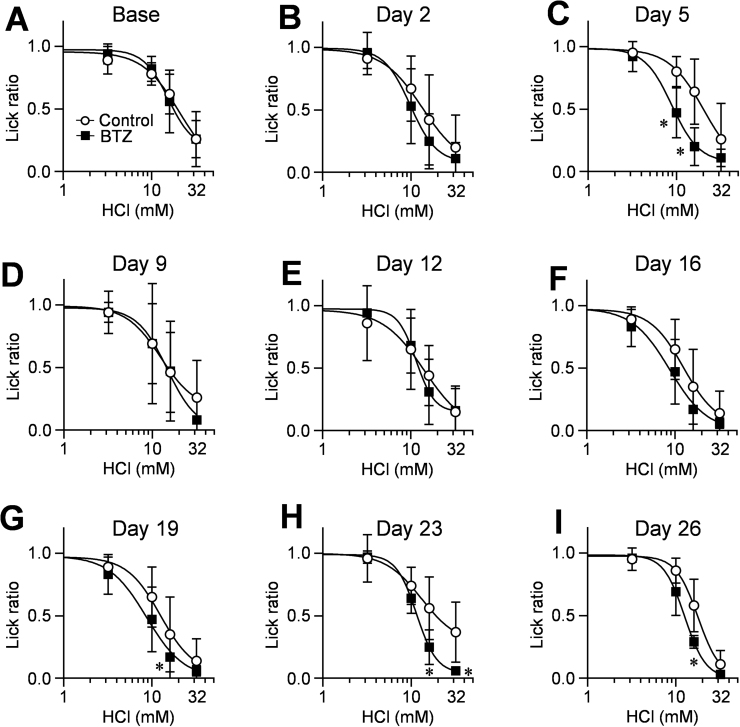
Behavioral responses to HCl solutions in bortezomib-administered mice. The lick ratios for 3.2–32 mM HCl (A–I) were determined in control and bortezomib-administered mice on day 0 (Base), 2, 5, 9, 12, 16, 19, 23 and 26. Concentrations of HCl solutions were 3.2, 10, 16 and 32 mM. Each point represents the mean ± SD (N = 4–10). *: *p* < 0.05 vs control in multiple comparison of Bonferroni test. BTZ: bortezomib.

**Fig. 4:**
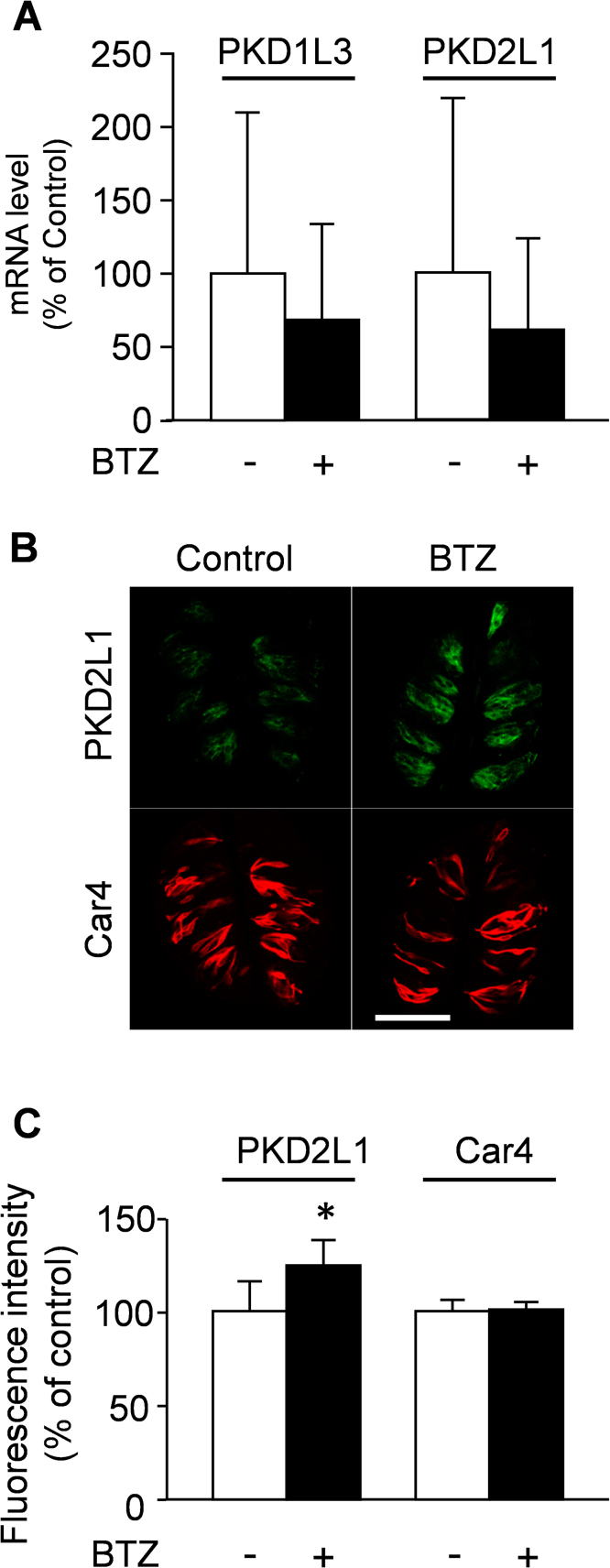
Expression levels of PKD1L3 and PKD2L1 in the CP of bortezomib-administered mice. (A) Quantification of the mRNA expression of PKD1L3 and PKD2L1 in the CP of control and bortezomib-administered mice on day 26 was performed by real-time PCR. Each bar represents the mean ± SD (N = 5 − 7). Panel B shows representative immunohistochemical images for PKD2L1 (green) and Car4 (red), the quantitative results for PKD2L1 and Car4-fluorescent intensity in CP being given in panel C. Scale bar = 100 μm. Each bar represents the mean ± SD (N = 5). *: p < 0.05 vs control. BTZ: bortezomib.

**Fig. 5:**
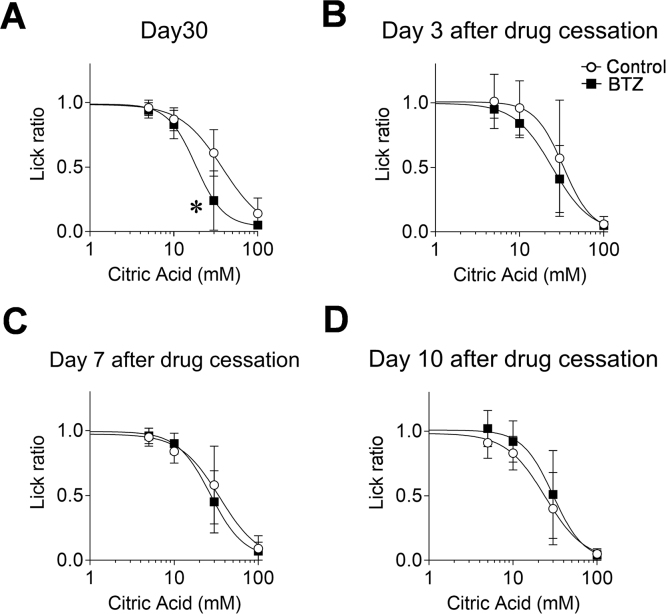
Behavioral response to citric acid solutions after the cessation of multiple bortezomib administration in mice. Mice were administered bortezomib or the vehicle following the schedule described under Materials and Methods up to day 36, and a brief-access test with 5, 10, 30 and 100 mM citric acid solutions was performed on day 30, and 3, 7 and 10 days after the final bortezomib administration. Each point represents the mean ± SD (N = 5–7). *: *p* < 0.05 vs control in multiple comparison of Bonferroni test. BTZ: bortezomib.

All data are expressed as means ± SD. To detect differences within and between the groups in response to the bortezomib administration and taste solutions, a two-way ANOVA was performed followed by a pairwise multiple-comparison procedure (Bonferroni test) to locate the significant difference indicated by ANOVA in brief-access test (Fig. 1, Fig. 2, Fig. 3, Fig. 4, Fig. 5). Pairwise comparisons were evaluated by means of the Mann-Whitney *U* test. A *p*-value of 0.05 or less was considered statistically significant.

## 3 Results

### 3.1 Five basic taste sensitivity

First, to determine whether taste sensitivity was affected or not, and when taste sensitivity alteration occurred by bortezomib administration, we assessed the sensitivity to five basic tastes in mice using the brief-access test (Fig. 1). As shown in Fig. 1C and D, statistical analysis by two-way ANOVA showed the significant decrease of lick ratios in the experiments of 5 and 10 mM citric acid solution in bortezomib-administered mice. On day 9, there was a significant increase of lick ratio in 5 mM citric acid solution (*p* = 0.033, CI: −0.552 to −0.018), but the increase was not continuous. We detected significant decrease of it on days 16 (5 mM: 0.86 vs 0.51, CI: 0.049–0.657, *p* = 0.018; 10 mM: 0.71 vs 0.32, CI: 0.084–0.692, *p* = 0.008), 19 (10 mM: 0.80 vs 0.44, CI: 0.130–0.597, *p* = 0.001) and 23 (5 mM: 0.79 vs 0.43, CI: 0.098–0.624, *p* = 0.004). Although significant changes in the lick ratios were also detected in other taste solutions in bortezomib-administered mice, tendency was not continuous, or in just one concentration of the taste solutions. Based on these findings, we assumed that multiple administration of bortezomib induced alteration in sour taste sensitivity. Thus, we used four-week schedule in following experiments.

### 3.2 Sour taste sensitivity

To assess the alteration of sour taste sensitivity in more detail, we performed an additional brief-access test using 2 series of sour tastants, citric acid and HCl. For both solutions, the lick ratios of mice for each solution in the control and bortezomib groups decreased concentration-dependently (Fig. 2, Fig. 3). A statistical analysis by two-way ANOVA showed the overall significance in lick ratios between control and bortezomib-administered mice in both sour taste solutions on days 23 (citric acid: *p* = 0.018, F(1, 15) = 7.08; HCl: *p* = 0.034, F(1, 10) = 6) and 26 (citric acid: *p* = 0.015, F(1, 11) = 8.36; HCl: *p* = 0.013, F(1, 11) = 8.82). A post hoc comparison test showed that the lick ratios in the bortezomib-administered mice for the 10 and 30 mM citric acid solutions, and 16 and/or 32 mM HCl ones were significantly less than those in control mice on days 19 (16 mM HCl: 0.55 vs 0.20, CI: 0.058–0.640, *p* = 0.012), 23 (10 mM citric acid: 0.86 vs 0.59, CI: 0.061–0.464, *p* < 0.01; 30 mM citric acid: 0.54 vs 0.27, CI: 0.061–0.465, *p* < 0.01; 16 mM HCl: 0.56 vs 0.25, CI: 0.085–0.535, *p* < 0.01; 32 mM HCl: 0.37 vs 0.06, CI: 0.078–0.528, *p* < 0.01) and 26 (10 mM citric acid: 0.87 vs 0.59, CI: 0.074–0.481, *p* < 0.01; 30 mM citric acid: 0.54 vs 0.21, CI: 0.127–0.533, *p* < 0.001; 16 mM HCl: 0.58 vs 0.29, CI: 0.104–0.480, *p* < 0.001), and the estimated IC_50_ values for bortezomib-administered mice were significantly lower than those for the control mice (Table 3, day23-citric acid: 16.4 vs 10.8, CI: 1.671–9.437, *p* < 0.01; day26-citric acid: 16.5 vs 11.9, CI: 0.779–8.490, *p* = 0.019; day26-HCl: 17.5 vs 12.3, CI: 1.443–9.048, *p* = 0.044), implying that bortezomib administration makes mice avoid drinking sour taste solutions at lower concentrations. Therefore, we concluded that multiple administration of bortezomib induced the increase of sour taste sensitivity, and day 26 was enough for development of this alteration.

**Table 3:** IC_50_ values for citric acid and HCl solutions in control and bortezomib-administered mice.

	(mM citric acid)		(mM HCl)	
	Control	Bortezomib	Control	Bortezomib
Baseline	16.21 ± 3.77	14.78 ± 2.95	23.02 ± 11.25	17.72 ± 6.53
Day 2	14.45 ± 3.93	9.63 ± 6.10	19.62 ± 17.50	10.16 ± 3.89
Day 5	14.45 ± 1.92	14.89 ± 4.56	15.13 ± 6.96	8.17 ± 2.35 *
Day 9	13.44 ± 2.34	12.52 ± 4.87	12.30 ± 6.97	12.46 ± 6.20
Day 12	11.17 ± 3.62	13.64 ± 4.45	12.65 ± 5.79	12.22 ± 2.78
Day 16	12.84 ± 5.26	13.04 ± 4.64	15.42 ± 8.59	9.39 ± 3.95
Day 19	12.69 ± 7.77	8.32 ± 2.57	22.79 ± 12.89	11.29 ± 2.57
Day 23	16.40 ± 2.39	10.85 ± 4.71 *	18.42 ± 12.82	11.57 ± 2.40
Day 26	16.52 ± 2.98	11.89 ± 2.97 *	17.51 ± 4.35	12.26 ± 1.87 *

Each value represents the mean ± SD (N = 4–12). *: *p* < 0.05 vs control on the corresponding day.

### 3.3 Expression levels of PKD1L3 and PKD2L1 in CP

It was reported that a heterotetramer of PKD1L3 and PKD2L1 plays an essential role in sour taste reception [19], [20], [21], [22], [23], [24]. Thus, the expression levels of them in the CP were determined. As shown in Fig. 4A, although the mRNAs for PKD1L3 and PKD2L1 were detected in the mouse CP, there was no difference in their expression levels on day 26 between control and bortezomib-administered mice. On immunohistochemistry, immunoreactivity for PKD2L1 was detected in taste buds of the CP, and its fluorescence intensity was significantly greater in bortezomib-administered mice compared to in control ones (Fig. 4B, C; 100.0% vs 124.3%, *p* = 0.028). On the other hand, the fluorescence intensity of Car4, a type III taste cell marker, was not altered (100.0% vs 100.2%, *p* = 0.754).

### 3.4 Reversibility of alteration of sour taste sensitivity

Finally, we examined whether the bortezomib-induced alteration in sour taste sensitivity is reversible or not. A statistical analysis by two-way ANOVA showed the overall significance in lick ratios between control and bortezomib-administered mice on day 30 (*p* = 0.027, F(1, 10) = 6.62). A post hoc comparison test showed that the lick ratios in the bortezomib-administered mice for 30 mM citric acid solution was significantly less than that in control mice on day 30 (0.61 vs 0.24, CI: 0.190–0.540, *p *< 0.0001). The IC_50_ value on day 30 also show significance between two groups (Table 4, 40.58 vs 20.48, CI: 1.565–41.755, *p* = 0.034), *i.e.*, a day after the administration. On the other hand, the lick ratios and IC_50_ values at 3, 7 and 10 days after cessation of the bortezomib administration were almost the same as comparable with them in the control mice.

**Table 4:** IC_50_ values for sour taste solutions after cessation of multiple bortezomib administration in mice.

	(mM citric acid)	
	Control	Bortezomib
Day 30	40.58 ± 22.37	20.48 ± 9.82 *
Day 3 after drug cessation	28.86 ± 12.76	26.94 ± 11.36
Day 7 after drug cessation	35.48 ± 15.26	30.62 ± 12.73
Day 10 after drug cessation	29.16 ± 18.48	30.42 ± 11.25

Each value represents the mean ± SD (N = 5–15). *: *p* *<* 0.05 vs control on the corresponding day.

## 4 Discussion

In this study, we made the following findings regarding the characteristics of bortezomib-induced taste disorder: (1) multiple administration of bortezomib induced an increase in sour taste sensitivity in mice, (2) bortezomib administration increased protein expression of PKD2L1, and (3) the altered sour taste sensitivity returned to the control level on cessation of its administration.

First, in brief-access tests using five basic taste solutions (Fig. 1), we detected significant increase or decrease in lick ratios in all of the tastes, suggesting the possibility that bortezomib changed the taste sensitivities on these tastes. Above all, we focused on the sour taste, because the significant changes were detected in both concentrations of solutions. We determined the lick ratios for 5 and 10 mM citric acid solutions in the two different sets of experiments as shown in Figs. 1C–D and 2, while the profiles of decrease in the lick ratios in the two concentrations were different. Since brief-access test is an evaluation method for behavior, motivation of mice for licking action is considered to be influenced by taste of solution which presented ahead. In fact, in the experiment of Fig. 1, mice licked sweet taste solution prior to the sour taste solution, whereas in the case of Fig. 2, they licked sour taste solution firstly. Furthermore, in the experiments with citric acid and HCl solutions shown in Fig. 2, Fig. 3, we detected overall significances in days 23 and 26. Collectively, we think that this discrepancy is acceptable and the repeated administration of bortezomib induces increase of sour taste sensitivity in mice.

Although taste sensitivity is thought to be different between mouse and human, they have one of the sour taste receptors, PKD2L1-PKD1L3, and we found the increased expression of PKD2L1 in taste buds in this study (Fig. 4). Thus, patients who administered bortezomib repeatedly might have increase in sour taste sensitivity, but to mention on this, detailed investigations are needed.

As in the cases of cyclophosphamide and oxaliplatin, there is a possibility that bortezomib might induce alterations in the morphology of taste buds and the number of type III taste cells, however, on HE staining and immunohistochemistry, there were no differences in the CP between control and bortezomib-administered mice on day 26 (Fig. S2). Based on these findings, it is suggested that the bortezomib-induced increase in sour taste sensitivity in mice might be due, at least in part, to the increase in PKD2L1 protein expression, this being supported by the findings that 3 days after bortezomib administration, there was no apparent alteration in the morphology of taste buds, the number of type III taste cells, or the mRNA and protein expression of PKD2L1 (data not shown). Therefore, it is suggested that the characteristics of the alteration in taste reception induced by anticancer drugs might differ individually based on their properties such as pharmacology, pharmacokinetics, *etc*.

Bortezomib inhibits the activity of proteasomes, which degrade poly-ubiquitinated proteins, and thus ubiquitinated proteins accumulate in the cells [25]. Ubiquitin acts as a marker for proteins undergoing degradation and also regulates trafficking of membrane proteins [26]. Among the four types of taste cells, type III taste cells are the only cells expressing protein gene product 9.5 [27], which can act as a ubiquitin C-terminal hydrolase [28], [29]. Therefore, there is a possibility that bortezomib administration might disrupt ubiquitin homeostasis in type III taste cells, and thus sour taste sensitivity might be altered. This hypothesis is supported by the finding that the expression level of PKD2L1 increased as to protein level, but not mRNA. To clarify this, detailed investigations are now in progress in our laboratory.

In this study, on the other hand, we revealed that the bortezomib-induced alteration of sour taste sensitivity had been reversed by 3 days after cessation of its administration (Fig. 5). The plasma half-life of bortezomib with a repeated administration protocol is reported to be 2–3 days (Drug label information for Velcade^®^). This information suggested that restoration of normal sour taste sensitivity might be due to a decrease in the bortezomib concentration in taste cells, and this is considered to support our hypothesis that bortezomib-induced taste disorder might be mediated by disruption of ubiquitin homeostasis, as mentioned above.

Acid stimulation is reported to stimulate the trigeminal sensory system as well as the taste reception system [30]. In addition, bortezomib administration induces peripheral neuropathy [11], [12]]. Therefore, there is a possibility that the behavioral change in the brief-access test might be caused by a change of peripheral sensory systems. Furthermore, some candidate sour receptor molecules have been reported, but the key factor for sour taste reception is still a matter of debate [23], [31]. Thus, further detailed investigation is needed, but PKD2L1 is considered to be one of the essential molecules in sour reception. On the other hand, Ishii et al. reported that capsaicin acts as an inhibitor of PKD1L3 and PKD2L1 [32], suggesting that in a clinical situation, pre-treatment with capsaicin might decrease the sensitivity to sour taste. Thus, there is a possibility that taking capsaicin-containing foods might ameliorate the bortezomib-induced increase in sour taste sensitivity.

## 5 Conclusion

In this study, we revealed that bortezomib administration increased PKD2L1 expression and sour taste sensitivity in mice. This taste disorder was reversed on cessation of its administration. We believe that these findings would help patients and medical staff by providing information on the characteristics of bortezomib-induced taste disorder.

## References

[1] Hesketh P.J., Grunberg S.M., Gralla R.J., Warr D.G., Roila F., de Wit R., Chawla S.P., Carides A.D., Ianus J., Elmer M.E., Evans J.K., Beck K., Reines S., Horgan K.J. (2003). The oral neurokinin-1 antagonist aprepitant for the prevention of chemotherapy-induced nausea and vomiting: a multinational, randomized, double-blind, placebo-controlled trial in patients receiving high-dose cisplatin-the Aprepitant Protocol 052 Study Group. J. Clin. Oncol..

[2] Hesketh P.J. (2008). Chemotherapy-Induced nausea and vomiting. N. Engl. J. Med..

[3] Zabernigg A., Gamper E.M., Giesinger J.M., Rumpold G., Kemmler G., Gattringer K., Sperner-Unterweger B., Holzner B. (2010). Taste alterations in cancer patients receiving chemotherapy: a neglected side effect. Oncologist.

[4] Halyard M.Y., Jatoi A., Sloan J.A., Bearden J.D., Vora S.A., Atherton P.J., Perez E.A., Soori G., Zalduendo A.C., Zhu A., Stella P.J., Loprinzi C.L. (2007). Does zinc sulfate prevent therapy-induced taste alterations in head and neck cancer patients? Results of phase III double-blind, placebo-controlled trial from the North Central Cancer Treatment Group (N01C4). Int. J. Radiat. Oncol. Biol. Phys..

[5] Mukherjee N., Delay E.R. (2011). Cyclophosphamide-induced disruption of umami taste functions and taste epithelium. Neuroscience.

[6] Mukherjee N., Carroll B.L., Spees J.L., Delay E.R. (2013). Pre-treatment with amifostine protects against cyclophosphamide-induced disruption of taste in mice. PLoS One.

[7] Ohishi A., Nishida K., Yamanaka Y., Miyata A., Ikukawa A., Yabu M., Miyamoto K., Bansho S., Nagasawa K. (2016). Oxaliplatin alters expression of T1R2 receptor and sensitivity to sweet taste in rats. Biol. Pharm. Bull..

[8] Williamson M.J., Silva M.D., Terkelsen J., Robertson R., Yu L., Xia C., Hatsis P., Bannerman B., Babcock T., Cao Y., Kupperman E. (2009). The relationship among tumor architecture, pharmacokinetics, pharmacodynamics, and efficacy of bortezomib in mouse xenograft models. Mol. Cancer Ther..

[9] Richardson P.G., Sonneveld P., Schuster M.W., Irwin D., Stadtmauer E.A., Facon T., Harousseau J.L., Ben-Yehuda D., Lonial S., Goldschmidt H., Reece D., San-Miguel J.F., Bladé J., Boccadoro M., Cavenagh J., Dalton W.S., Boral A.L., Esseltine D.L., Porter J.B., Schenkein D., Anderson K.C. (2005). Bortezomib or high-dose dexamethasone for relapsed multiple myeloma. N. Engl. J. Med..

[10] Fanucchi M.P., Fossella F.V., Belt R., Natale R., Fidias P., Carbone D.P., Govindan R., Raez L.E., Robert F., Ribeiro M., Akerley W., Kelly K., Limentani S.A., Crawford J., Reimers H.J., Axelrod R., Kashala O., Sheng S., Schiller J.H. (2006). Randomized phase II study of bortezomib alone and bortezomib in combination with docetaxel in previously treated advanced non-small-cell lung cancer. J. Clin. Oncol..

[11] Bruna J., Udina E., Alé A., Vilches J.J., Vynckier A., Monbaliu J., Silverman L., Navarro X. (2010). Neurophysiological, histological and immunohistochemical characterization of bortezomib-induced neuropathy in mice. Exp. Neurol..

[12] Carozzi V.A., Renn C.L., Bardini M., Fazio G., Chiorazzi A., Meregalli C., Oggioni N., Shanks K., Quartu M., Serra M.P., Sala B., Cavaletti G., Dorsey S.G. (2013). Bortezomib-induced painful peripheral neuropathy: an electrophysiological, behavioral, morphological and mechanistic study in the mouse. PLoS One.

[13] Papandreou C.N., Daliani D.D., Nix D., Yang H., Madden T., Wang X., Pien C.S., Millikan R.E., Tu S.M., Pagliaro L., Kim J., Adams J., Elliott P., Esseltine D., Petrusich A., Dieringer P., Perez C., Logothetis C.J. (2004). Phase I trial of the proteasome inhibitor bortezomib in patients with advanced solid tumors with observations in androgen-independent prostate cancer. J. Clin. Oncol..

[14] Ogawa Y., Tobinai K., Ogura M., Ando K., Tsuchiya T., Kobayashi Y., Watanabe T., Maruyama D., Morishima Y., Kagami Y., Taji H., Minami H., Itoh K., Nakata M., Hotta T. (2008). Phase I and II pharmacokinetic and pharmacodynamic study of the proteasome inhibitor bortezomib in Japanese patients with relapsed or refractory multiple myeloma. Cancer Sci..

[15] Murata Y., Nakashima K., Yamada A., Shigemura N., Sasamoto K., Ninomiya Y. (2003). Gurmarin suppression of licking responses to sweetener-quinine mixtures in C57BL mice. Chem. Senses.

[16] Yoshida R., Ohkuri T., Jyotaki M., Yasuo T., Horio N., Yasumatsu K., Sanematsu K., Shigemura N., Yamamoto T., Margolskee R.F., Ninomiya Y. (2010). Endocannabinoids selectively enhance sweet taste. Proc. Natl. Acad. Sci. U. S. A..

[17] Kusuhara Y., Yoshida R., Ohkuri T., Yasumatsu K., Voigt A., Hübner S., Maeda K., Boehm U., Meyerhof W., Ninomiya Y. (2013). Taste responses in mice lacking taste receptor subunit T1R1. J. Physiol..

[18] Nishida K., Kitada T., Kato J., Dohi Y., Nagasawa K. (2013). Expression of equilibrative nucleoside transporter 1 in rat circumvallate papillae. Neurosci. Lett..

[19] Lopez-Jimenez N.D., Cavenagh M.M., Sainz E., Cruz-Ithier M.A., Battey J.F., Sullivan S.L. (2006). Two members of the TRPP family of ion channels, Pkd1l3 and Pkd2l1, are co-expressed in a subset of taste receptor cells. J. Neurochem..

[20] Ishimaru Y., Inada H., Kubota M., Zhuang H., Tominaga M., Matsunami H. (2006). Transient receptor potential family members PKD1L3 and PKD2L1 form a candidate sour taste receptor. Proc. Natl. Acad. Sci..

[21] Huang A.L., Chen X., Hoon M.A., Chandrashekar J., Guo W., Tränkner D., Ryba N.J., Zuker C.S. (2006). The cells and logic for mammalian sour taste detection. Nature.

[22] Kataoka S., Yang R., Ishimaru Y., Matsunami H., Sévigny J., Kinnamon J.C., Finger T.E. (2008). The candidate sour taste receptor, PKD2L1, is expressed by type III taste cells in the mouse. Chem. Senses.

[23] Horio N., Yoshida R., Yasumatsu K., Yanagawa Y., Ishimaru Y., Matsunami H., Ninomiya Y. (2011). Sour taste responses in mice lacking PKD channels. PLoS One.

[24] Yu Y., Ulbrich M.H., Li M.H., Dobbins S., Zhang W.K., Tong L., Isacoff E.Y., Yang J. (2012). Molecular mechanism of the assembly of an acid-sensing receptor ion channel complex. Nat. Commun..

[25] Fang J., Rhyasen G., Bolanos L., Rasch C., Varney M., Wunderlich M., Goyama S., Jansen G., Cloos J., Rigolino C., Cortelezzi A., Mulloy J.C., Oliva E.N., Cuzzola M., Starczynowski D.T. (2012). Cytotoxic effects of bortezomib in myelodysplastic syndrome/acute myeloid leukemia depend on autophagy-mediated lysosomal degradation of TRAF6 and repression of PSMA1. Blood.

[26] Terada K., Horinouchi T., Fujioka Y., Higashi T., Nepal P., Horiguchi M., Karki S., Hatate C., Hoshi A., Harada T., Mai Y., Ohba Y., Miwa S. (2014). Agonist-promoted ubiquitination differentially regulates receptor trafficking of endothelin type A and type B receptors. J. Biol. Chem..

[27] Yee C.L., Yang R., Böttger B., Finger T.E., Kinnamon J.C. (2001). Type III cells of rat taste buds: immunohistochemical and ultrastructural studies of neuron-specific enolase, protein gene product 9.5, and serotonin. J. Comp. Neurol..

[28] Larsen C.N., Price J.S., Wilkinson K.D. (1996). Substrate binding and catalysis by ubiquitin C-terminal hydrolases: identification of two active site residues. Biochemistry.

[29] Larsen C.N., Krantz B.A., Wilkinson K.D. (1998). Substrate specificity of deubiquitinating enzymes: ubiquitin C-terminal hydrolases. Biochemistry.

[30] Bryant B.P., Moore P.A. (1995). Factors affecting the sensitivity of the lingual trigeminal nerve to acids. Am. J. Physiol..

[31] Ye W., Chang R.B., Bushman J.D., Tu Y.H., Mulhall E.M., Wilson C.E., Cooper A.J., Chick W.S., Hill-Eubanks D.C., Nelson M.T., Kinnamon S.C., Liman E.R. (2016). The K+ channel KIR2: 1 functions in tandem with proton influx to mediate sour taste transduction. Proc. Natl. Acad. Sci. U. S. A..

[32] Ishii S., Kurokawa A., Kishi M., Yamagami K., Okada S., Ishimaru Y., Misaka T. (2012). The response of PKD1L3/PKD2L1 to acid stimuli is inhibited by capsaicin and its pungent analogs. FEBS J..

