# Supplementary figures and images for: Bortezomib alters sour taste sensitivity in mice
Source: Toxicol Rep. 2017 Mar 10;4:172–80. doi: 10.1016/j.toxrep.2017.03.003 (PMC5615125; doi:10.1016/j.toxrep.2017.03.003)

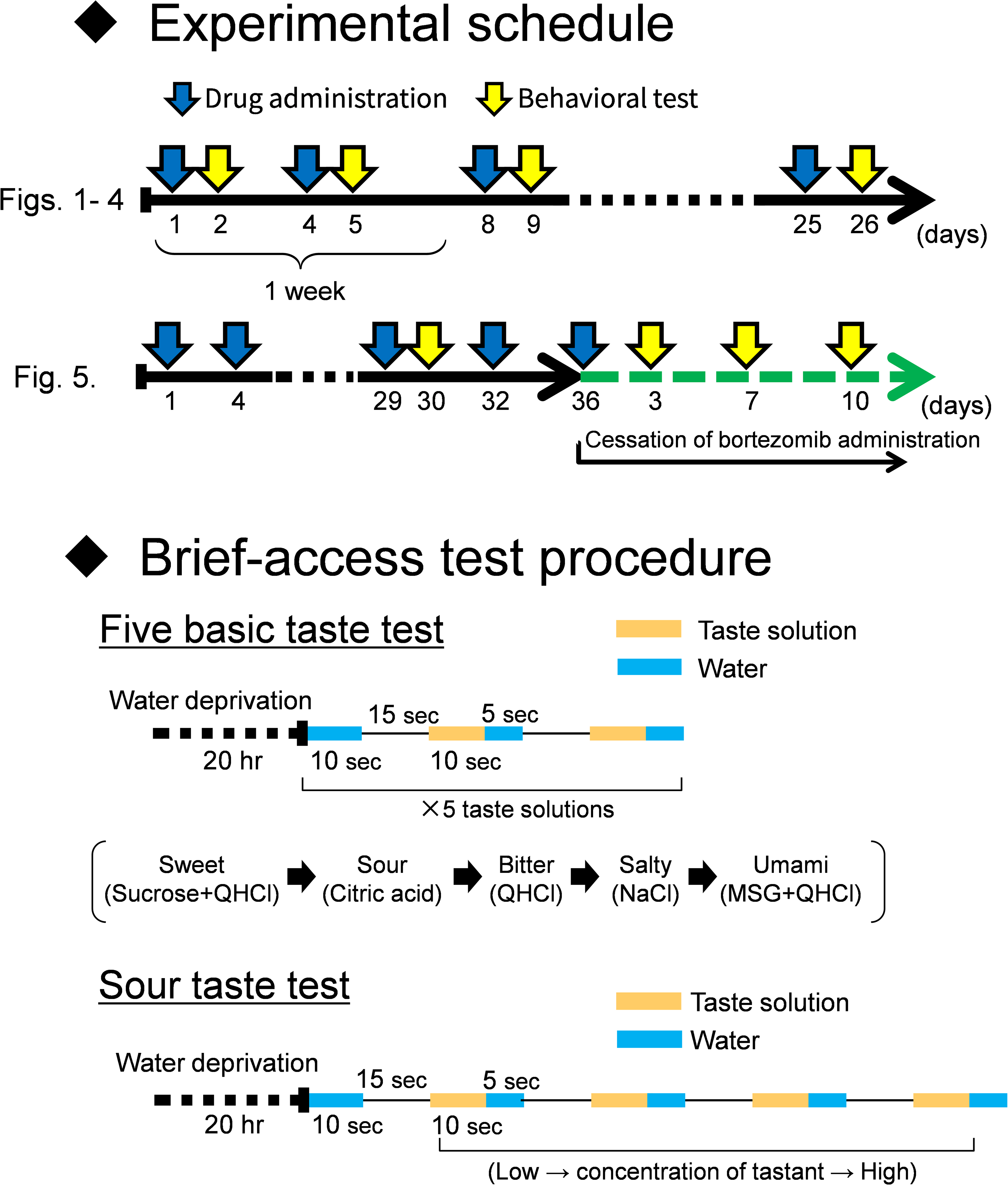

Supplement: Supplementary file 1 [file mmc1.jpg]

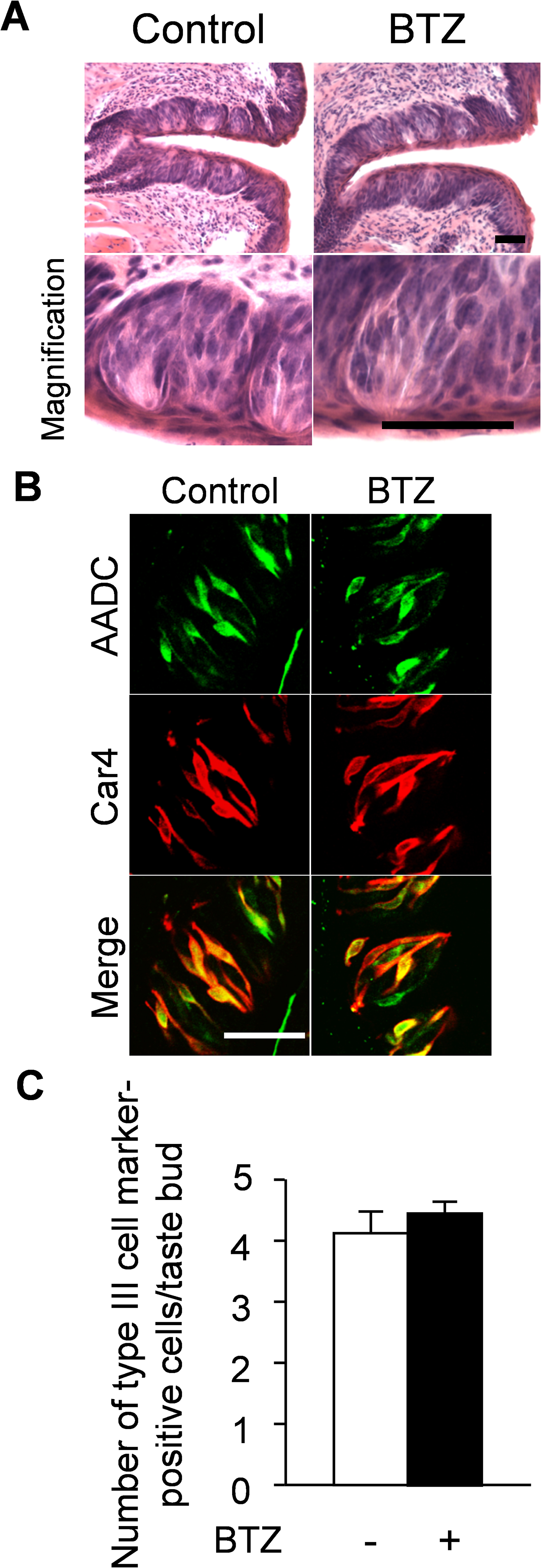

Supplement: Supplementary file 2 [file mmc2.jpg]
