# Supplementary material for: Bortezomib alters sour taste sensitivity in mice
Source: Toxicol Rep. 2017 Mar 10;4:172–80. doi: 10.1016/j.toxrep.2017.03.003 (PMC5615125; doi:10.1016/j.toxrep.2017.03.003)
Supplement: Supplementary file 3 [file mmc3.docx]

**Supplementary material**

**Figure legends**

**Fig. S1. Schematic images of experimental schedules and brief-access tests.**

**Fig. S2. Morphology of taste buds and number of type III taste cells in the CP of bortezomib-administered mice on day 26.**

(A) Representative photomicrographs of HE staining of the CP of control and bortezomib-administered mice. Scale bar = 50 μm. (B, C) Type III taste cells in the CP of control and bortezomib-administered mice. Panel B shows representative immunohistochemical images for Car4 (red) and AADC (green), respectively, the quantitative results being given in panel C. The antibody used for detection of AADC is summarized in Supplementary Table S2. Scale bar = 100 μm. Each bar represents the mean ± SD (N = 4-5). BTZ: bortezomib.

**Supplementary** **tables**

**Table S1. pH of each sour taste solution used in brief-access tests.**

| Citric acid (mM) | 5 | 10 | 30 | 100 | 300 |
| --- | --- | --- | --- | --- | --- |
| pH | 2.78 | 2.70 | 2.58 | 2.40 | 2.14 |
| Hydrochloric acid (mM) | 3.2 | 10 | 16 | 32 | 100 |
| pH | 2.94 | 2.59 | 2.44 | 2.20 | 1.78 |

**Table S2. Antibody used for immunohistochemistry.**

| Antigen | Primary Ab | Secondary Ab |
| --- | --- | --- |
| AADC | Rabbit polyclonal Ab (1:200; BML-AZ1030-0050, Enzo) | Donkey anti-rabbit IgG conjugated with Alexa Fluor^®^ 488 (1:1000; A21206, Life Technologies) |
